# Caffeic Acid Acts as a Potent Senomorphic and Alleviates Inflammation and Lung Fibrosis by Covalently Targeting Annexin A5 Protein in Mice

**DOI:** 10.1002/EXP.20240069

**Published:** 2025-12-12

**Authors:** Yinhua Zhu, Ying Zhang, Qianyu Zhang, Ping Song, Junzhe Zhang, Ang Ma, Chen Wang, Peng Gao, Tong Yang, Lirun Zhou, Qiaoli Shi, Yin Kwan Wong, Yongting Luo, Huan Tang, Jigang Wang

**Affiliations:** ^1^ Beijing Advanced Innovation Center for Food Nutrition and Human Health, Department of Nutrition and Health China Agricultural University Beijing China; ^2^ State Key Laboratory For Quality Ensurance and Sustainable Use of Dao‐di Herbs, Artemisinin Research Center, and Institute of Chinese Materia Medica China Academy of Chinese Medical Sciences Beijing China; ^3^ Department of Biological Sciences National University of Singapore Singapore

**Keywords:** annexin A5, caffeic acid, SASP, senescent cells, senomorphics

## Abstract

The accumulation of senescent cells and their secretion of senescence‐associated secretory phenotype (SASP) play important roles in the pathogenesis of idiopathic pulmonary fibrosis (IPF). Small molecules, known as senolytics or senomorphics, have been effective in targeting senescent cells. Although senolytic drugs have been well‐studied in pulmonary fibrosis, senomorphics with defined protein targets and potential applications are rarely investigated. In this study, we identified a widely sourced natural product, caffeic acid (CA), to act as a potent senomorphic that effectively inhibits the secretion of SASP in senescent lung cells. We demonstrated that the covalent binding of CA to Annexin A5 protein triggered its degradation, PKCθ deactivation, and the inhibition of the NF‐κB inflammatory pathway in senescent cells. Notably, CA exhibited a promising effect in limiting inflammation in the lung and circulatory system, alleviating pulmonary pathology, and improving physical function in a bleomycin‐induced pulmonary fibrosis mouse model. Our investigation suggests that Annexin A5 could be used as the target for the precise intervention of aging‐related diseases such as IPF.

## Graphical abstract

1. Caffeic acid (CA) is a novel senomorphic.

2. Annexin A5 is the direct protein target of CA in senescent cells.

3. The covalent binding of CA to Annexin A5 protein triggers its degradation, PKCθ deactivation, and the inhibition of the NF‐κB inflammatory pathway.

4. CA effectively alleviates inflammation and lung fibrosis in mouse model.

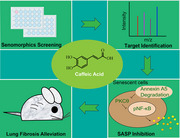

## 1 Introduction

Idiopathic pulmonary fibrosis (IPF) is a chronic, progressive, fibrotic interstitial lung disease that is more prevalent in the elderly population [1, 2]. Currently, two drugs, pirfenidone and nintedanib, have received approval from the Food and Drug Administration (FDA) for clinical treatment of IPF to delay disease progression [3]. However, these two drugs only alleviate the progression of IPF to a limited degree and have side effects, such as liver injury [3]. Therefore, developing safe and effective intervention strategies is essential to prevent or treat pulmonary fibrosis.

Recently, mounting evidence indicates that the accumulation of senescent cells and their secretion of senescence‐associated secretory phenotype (SASP) contribute to the pathogenesis of IPF [4, 5]. Correspondingly, targeting senescent cells with small molecules has emerged as a promising approach to overcome lung fibrosis [6]. The reported chemical approaches to regulate senescent cells include senolytics, a class of small molecules that can selectively eliminate senescent cells; or senomorphics, which modulate senescent cells by inhibiting SASP secretion without killing the senescent cells [7, 8, 9, 10]. Two widely used senolytics are the combination of Dasatinib (D) + Quercetin (Q) and the dual inhibitor of Bcl2 and BclxL ABT263, both of which have satisfactory therapeutic effects in bleomycin‐induced pulmonary fibrosis mouse models and D+Q were even tested in clinical trials [4, 11, 12, 13]. However, the potential toxicity and off‐target effect of current senolytics have raised concerns about their further development. The alternative approach using senomorphics to inhibit SASP secretion without killing senescent cells is safer, although their roles in the intervention of pulmonary fibrosis have rarely been reported. Notably, there is a significant lack of senomorphics with defined protein targets and applications.

In this study, we identified potent senomorphics from natural products and found that caffeic acid (CA) could effectively inhibit the secretion of SASP in senescent lung cells. Further mechanistic studies determined that covalent binding of CA to Annexin A5 (ANXA5) protein resulted in protein degradation, eliciting PKCθ deactivation and the inhibition of the NF‐κB inflammatory pathway in senescent cells. CA exhibited a promising effect in limiting inflammation in the lung and circulatory system, alleviating pulmonary pathology, as well as improving physical function in a bleomycin‐induced pulmonary fibrosis mouse model. Our study identifies CA as a promising senomorphic and suggests that ANXA5 could be a possible target for precise intervention in aging‐related diseases like IPF.

## 2 Results

### 2.1 Caffeic Acid was Screened as a Potent Senomorphic

**FIGURE 1:**
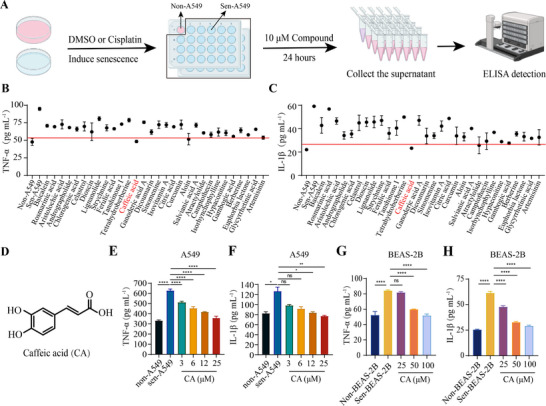
CA was screened as a novel senomorphic. (A) Flow diagram for the screening of senomorphics from natural products, *n* = 3. (B,C) Detection of TNF‐α (B) or IL‐1β (C) in the culture medium of senescent A549 cells treated with 10 µm compound for 24 h, *n* = 3. (D) Chemical structure of CA. (E,F) CA dose‐dependently decreased the concentration of TNF‐α (E) or IL‐1β (F) in the culture medium of senescent A549 cells, *n* = 3. (G,H) Changes in the concentrations of TNF‐α (G) or IL‐1β (H) in the culture medium of senescent BEAS‐2B cells treated with increasing dosages of CA, *n* = 3. Data are the mean ± s.e.m. For (E–H), a one‐way ANOVA test was used. *P < 0.05, **P < 0.01, ***P < 0.001, ****P < 0.0001. ns indicates no significance.

In our previous study, we collected a small pool of active ingredients from traditional Chinese medicine (TCM) and established a senescent lung cell model to screen for senolytics [14]. In this study, to identify senomorphics from natural products, we expanded our compound library by adding common components from food and beverages. We performed screening using the following system (Figure 1A and Table S1): Senescence was induced in A549 cells using cisplatin treatment and characterized by senescence‐associated beta‐galactosidase (SA‐β‐gal) staining (Figure S1A). After the senescent cells were incubated with 10 µm of the compounds for 24 h, the content of three representative secreted inflammatory factors (TNF‐α, IL‐6, and IL‐1β) in the cell culture medium was measured by ELISA to assess the inhibitory effect of these compounds on SASP secretion. Among the tested compounds, caffeic acid (CA), a natural product with anti‐oxidant and anti‐inflammatory activity and widely found in food and traditional Chinese medicine [15, 16], was found to efficiently reduce the secretion of the three cytokines to the control level (Figure 1B–D and Figure S1B). As CA exhibited the optimal SASP‐inhibitory activity in our screening (Table S1), it was chosen for subsequent analyses.

Next, we examined if CA inhibited SASP secretion in a dosage and cell‐type‐dependent manner. The results indicated that beginning at a dosage of 3 µm, CA exhibited significant inhibitory activity in senescent A549 cells (Figure 1E,F and Figure S1C). Moreover, the levels of the three inflammatory factors in the cell culture medium gradually decreased with increasing CA concentrations, suggesting a dose‐dependent inhibitory effect of CA (Figure 1E,F and Figure S1C). Despite the higher concentrations of CA required, similar results were also obtained in another human lung epithelial cell (BEAS‐2B) senescence model (Figure 1G,H and Figure S1D). Notably, CA did not affect the cell viability of senescent A549 cells even at 150 µm (Figure S1E). In conclusion, the above results demonstrated that caffeic acid was a potent senomorphic that could inhibit SASP secretion in different types of senescent cells.

### 2.2 Annexin A5 was Identified as a Covalent Target of CA in Senescent Cells

**FIGURE 2:**
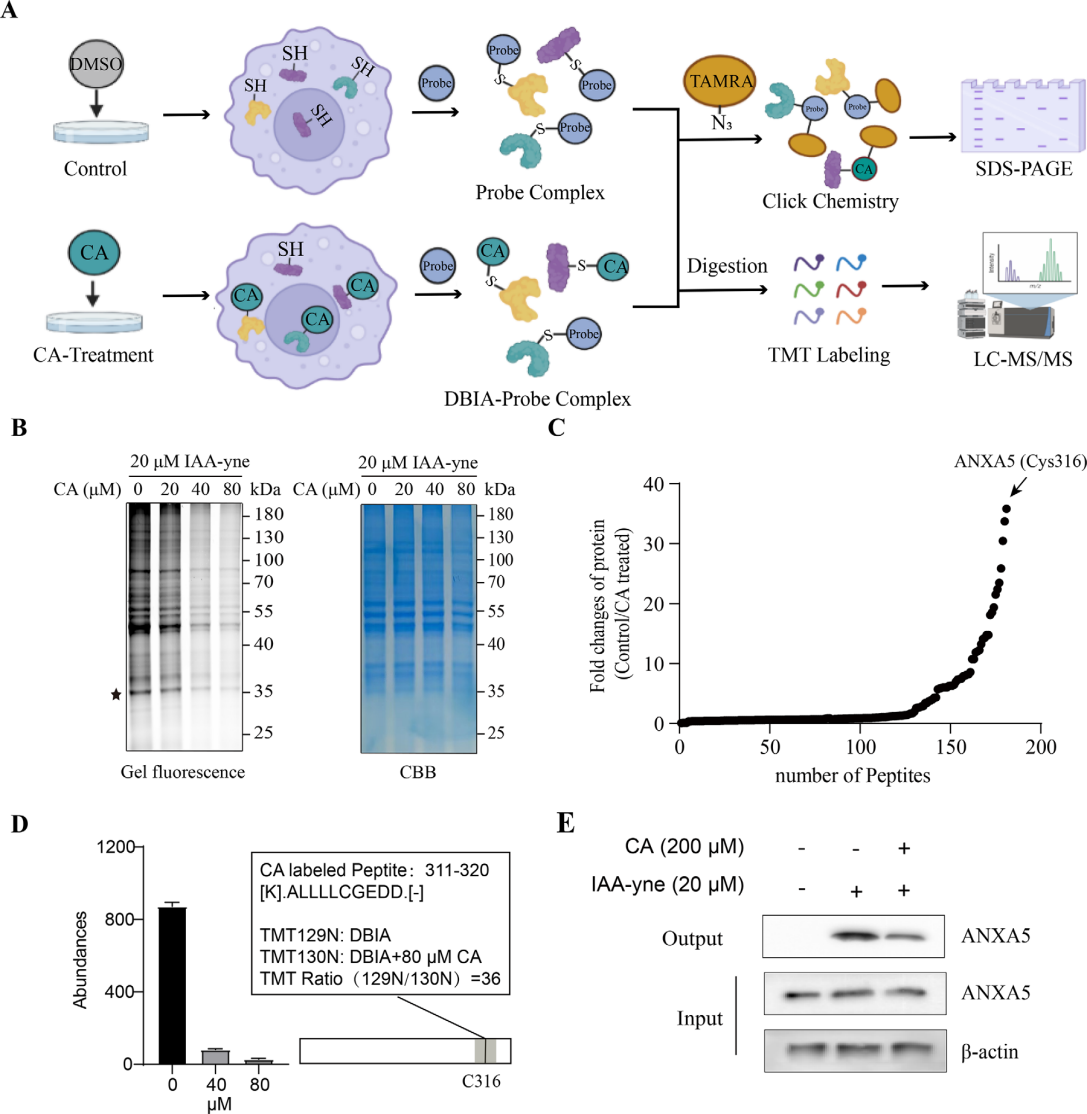
Annexin A5 was identified as a covalent target of CA in senescent cells. (A) Flow diagram of target identification of CA in senescent A549 cells by ABPP. (B) CA competes with the IAA probe for binding to the cysteine residues of proteins in senescent A549 cells. Left, in‐gel fluorescence. Right, coomassie brilliant blue (CBB) staining. The asterisk denotes the position of the target used in subsequent validation. (C) Protein targets of CA identified by ABPP in senescent A549 cells. (D) High concentrations of CA compete with DBIA for labeling to ANXA5 via the Cys316 residue. (E) CA interacts with ANXA5 in cells, as confirmed by pull‐down assay.

To explore the mechanism of action of CA as a senomorphic, we performed global profiling for the direct protein target of CA in senescent cells. CA's chemical structure has a typical unsaturated ketone group [17], potentially serving as a Michael addition receptor to covalently react with the thiol group of cysteine residues in proteins. Therefore, we employed competitive activity‐based protein profiling (ABPP) technology to identify the potential protein targets as previously reported [14, 18, 19, 20]. As presented in the flowchart (Figure 2A), we tested the ability of CA to covalently bind to the cysteine residues of cellular proteins. We determined that CA could effectively compete away fluorescent signals of proteins labeled by the iodoacetamide probe (Figure 2B), a well‐known cysteine blocker, confirming our inference above.

We then performed a streamlined cysteine activity‐based protein profiling to identify proteome‐wide targets of CA using a desthiobiotin iodoacetamide (DBIA) probe [19]. We generated a cysteinome dataset demonstrating specific cysteine residues bound to CA as highlighted by the competitive ratio in DBIA probe‐mediated enrichment (Figure 2C). Among the cysteines that interacted with CA, Cys316 from ANXA5 was the most competitive, with a competitive ratio of 36 compared to the control group (Figure 2C,D). ANXA5 is a widely distributed member of the calcium and phospholipid binding protein family, engaged in various processes like inflammatory response, cellular senescence, and apoptosis [21, 22]. We performed the pull‐down assay and observed that CA reduced ANXA5 protein enrichment in IAA probe‐treated samples (Figure 2E). Taken together, these findings suggested that ANXA5 is a potential target of CA in senescent cells.

### 2.3 CA Directly Targeted ANXA5 Through Cys316

**FIGURE 3:**
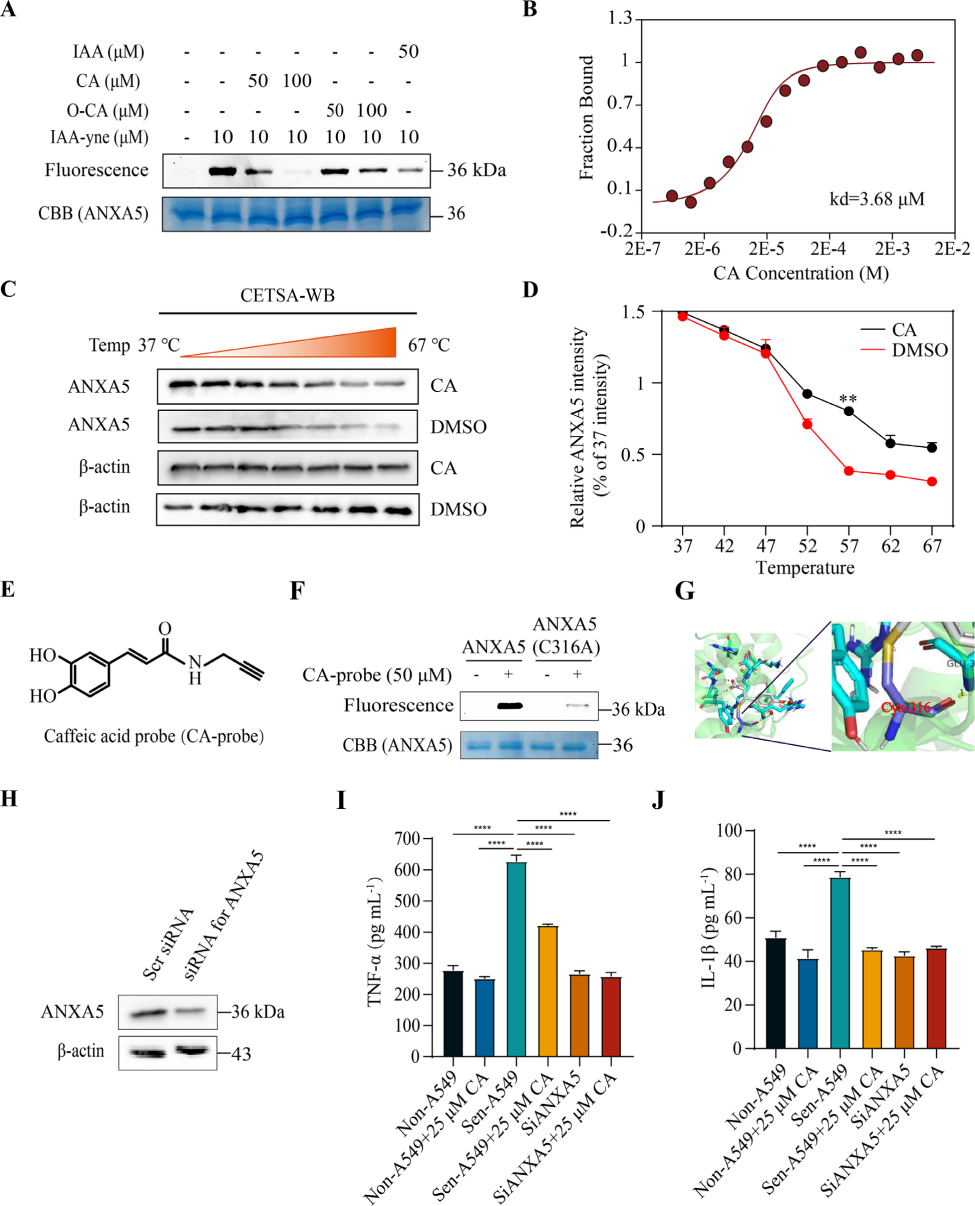
CA directly targeted ANXA5 through Cys316. (A) CA or its oxidized form competes with the IAA probe for binding to the purified recombinant ANXA5 as characterized by in‐gel fluorescence assay. (B) Measurement of the binding affinity between CA and ANXA5 protein by MST assay. (C, D) Detection of the interaction between CA and ANXA5 by CETSA. Left (C), detection of ANXA5 or β‐actin protein by Western blot. Right (D), quantitative statistics of the Western blot result. (E) Chemical structure of the CA probe. (F) CA probe mediated‐labeling of wild‐type or C316A mutant recombinant ANXA5 detected using in‐gel fluorescence assay. (G) Binding sites of CA (gray sticks) in ANXA5 (PDB code: 1ANW) simulated by molecular docking. (H) Detection of the siRNA‐mediated knockdown of ANXA5 in senescent A549 cells by Western blot. Scr siRNA, scrambled siRNA as a control. (I, J) Changes in the concentrations of TNF‐α (I) or IL‐1β (J) in the culture medium of non‐senescent or senescent A549 cells transfected with scrambled siRNA or ANXA5 siRNA following DMSO or 25 µM CA treatment, *n* = 3. Data are mean ± s.e.m. For (I,J), a one‐way ANOVA test was used; For (D), a two‐way ANOVA test was used. *P < 0.05, **P < 0.01, ***P < 0.001, ****P < 0.0001. ns indicates no significance.

Next, we designed a series of biochemical and cellular experiments to validate the binding of CA to ANXA5. First, recombinant ANXA5 protein was employed to test whether CA could compete with the IAA probe for labeling the protein in vitro. We found that 100 µm CA completely suppressed the fluorescence signal of ANXA5 labeled by a 10 µm IAA probe (Figure 3A). Notably, to exclude the possibility that the electron‐rich catechol moiety of CA was oxidized to electrophilic dopaquinone, responsible for the covalent binding to ANXA5 protein in cells as the previously reported binding mode [23], we prepared oxidized CA using tyrosinase. Interestingly, compared to native CA, the oxidized form of CA had an attenuated ability to compete with the IAA probe for binding proteins (Figure 3A), suggesting the functional role of its catechol moiety in binding to ANXA5. Second, we evaluated the binding affinity between CA and ANXA5 in solution as 3.68 µm through a micro‐scale thermophoresis (MST) assay (Figure 3B). Third, a cellular thermal shift assay (CETSA) indicated that CA significantly enhanced the thermal stability of ANXA5 but did not impact the control protein (Figure 3C,D), confirming the direct interaction between CA and ANXA5.

To validate the binding site of CA on ANXA5, we synthesized a caffeic acid probe (CA‐probe) (Figure 3E), which was well structurally characterized and exhibited a similar biological activity as CA in inhibiting SASP secretion in senescent cells (Figures S2–S5). We mutated the cysteine residue at position 316 to alanine and purified the mutant protein ANXA5 (C316A). The CA‐probe was used to label both the wild type and the point‐mutated protein. The results indicated that the wild‐type ANXA5 could be labeled by CA‐probe while the fluorescence signal on ANXA5 (C316A) dramatically decreased (Figure 3F). Furthermore, our molecular docking findings indicated that CA could form a covalent bond with Cys316 on ANXA5 (Figure 3G). Finally, a corresponding siRNA was used to knock down the expression of ANXA5 in A549 cells, and a Western blot confirmed the knockdown efficiency (Figure 3H). We found that the senescent A549 cells had increased TNF‐α and IL‐1β secretion relative to control cells, whereas knockdown of ANXA5, as well as CA treatment, inhibited the secretion of the two inflammatory factors (Figure 3I,J). Critically, CA treatment could not enhance the inhibitory effect in senescent cells with decreased expression of ANXA5 (Figure 3I,J), indicating that the function of CA relied on ANXA5. Taken together, the above data suggest that CA inhibited the SASP secretion in senescent cells by targeting ANXA5.

### 2.4 CA Induced ANXA5 Protein Degradation to Inhibit NF‐κB Activation in Senescent Cells

**FIGURE 4:**
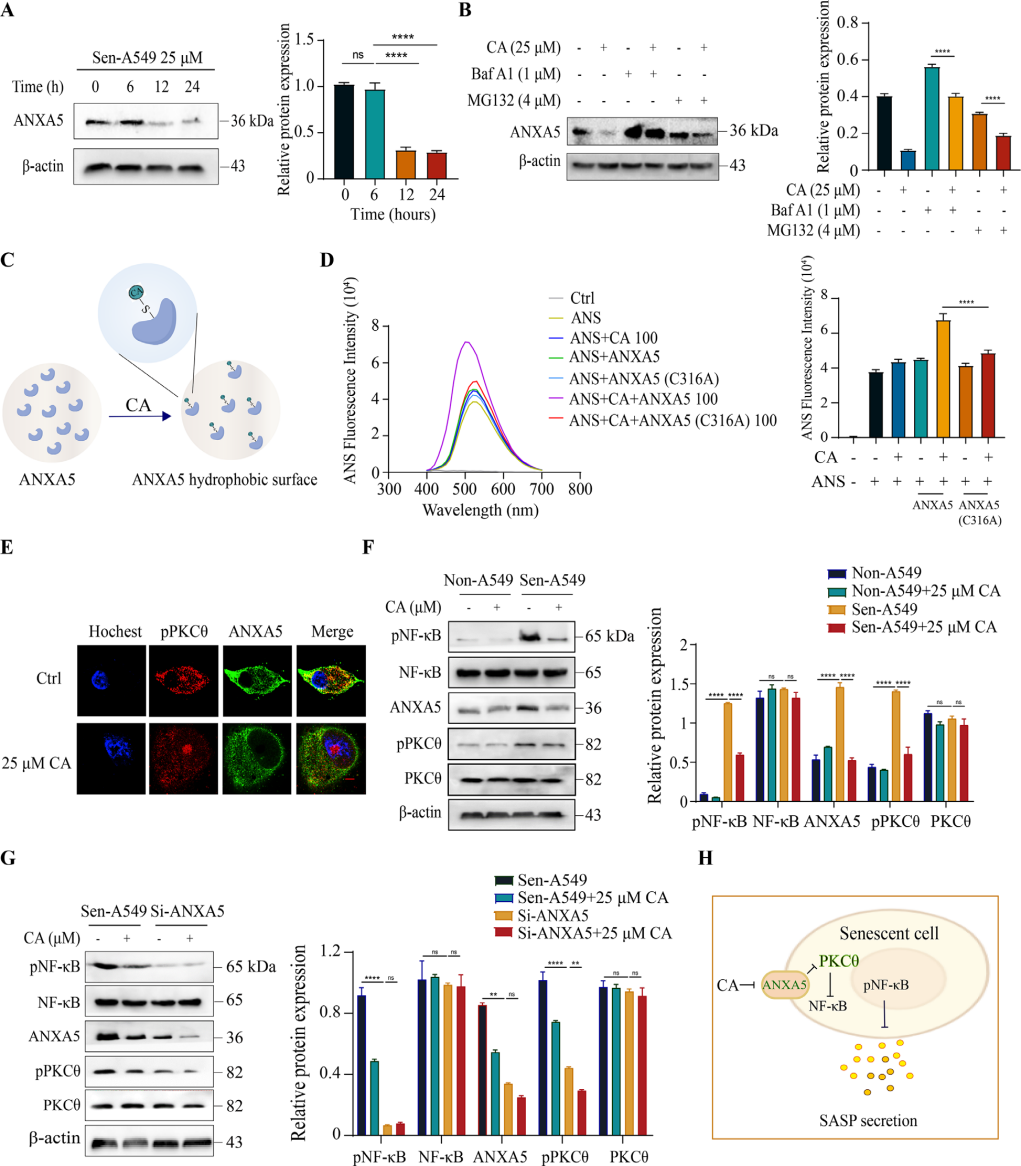
CA induced ANXA5 protein degradation to inhibit NF‐κB activation in senescent cells. (A) Detection of ANXA5 protein levels in senescent A549 cells at different time points following 25 µm CA treatment. Left, detection of ANXA5 protein by Western blot. Right, quantitative statistics of the Western blot result, *n* = 3. (B) The effect of BAF A1 and MG132 treatment on CA‐induced ANXA5 protein degradation in senescent A549 cells. Left, analysis of the ANXA5 via Western blot. Right, quantitative statistics of the Western blot result, *n* = 3. (C) Schematic representation of the covalent binding of CA to ANXA5 protein resulting in conformational changes. (D) The binding of the ANS probe to wild‐type or C316A mutant recombinant ANXA5 after CA treatment as assessed by the ANS fluorescence assay. Left, fluorescence spectra change. Quantitative statistics are presented on the right, *n* = 3. (E) Detection of ANXA5 or pPKCθ protein levels in senescent A549 cells treated with DMSO or CA by immunofluorescence. Scale bar, 100 µm. (F) Changes in protein levels of ANXA5, NF‐κB, and PKCθ or their phosphorylated form in non‐senescent or senescent A549 cells following DMSO or 25 µm CA treatment. Left, analyzing proteins by Western blot. Quantitative statistics are presented on the right, *n* = 3. (G) Detection of ANXA5, NF‐κB, PKCθ, or their phosphorylated form in senescent A549 cells transfected with scrambled siRNA or ANXA5 siRNA following DMSO or 25 µm CA treatment. The Western blot results are presented on the left. Quantitative statistics are presented on the right, *n* = 3. (H) Schematic diagram of CA reducing SASP secretion by inducing ANXA5 protein degradation, inhibiting PKCθ and NF‐κB activation in senescent cells. Data are the mean ± s.e.m. For (A, B, and D), a one‐way ANOVA test was used. For (F and G), a two‐way ANOVA test was used. *P < 0.05, **P < 0.01, ***P < 0.001, ****P < 0.0001. ns indicates no significance.

We attempted to characterize the direct interaction between CA and ANXA5 and its effect on protein function and downstream signal transduction in senescent cells. We evaluated whether the protein abundance of ANXA5 was affected by CA treatment. The Western blot results showed that the protein level of ANXA5 significantly decreased with increasing concentration and time of CA treatment (Figure 4A and Figure S6). Due to the pronounced effect of CA treatment at 25 µm, this concentration was selected for subsequent experiments. We used MG132 or Bafilomycin A1 (BAF A1) to inhibit the two major protein degradation pathways, primarily the ubiquitin‐proteasome system (UPS) and the autophagic‐lysosomal pathway (ALP) [24]. The results indicated that treatment with BAF A1 but not MG132 blocked the CA‐induced ANXA5 degradation and caused protein accumulation (Figure 4B), suggesting that ANXA5 is degraded via autophagy. Therefore, we hypothesized that the covalent binding of CA to ANXA5 protein caused conformational changes, such as hydrophobic exposure, leading to its degradation, as depicted in Figure 4C. To validate the hypothesis, we used a fluorescent dye 1‐anilinonaphthalene‐8‐sulfonic acid (ANS) that could bind to the protein hydrophobic regions to assess the potential impact of CA on the conformation of ANXA5 as previously reported [25]. Compared to the control group containing ANS and wild‐type ANXA5 protein, adding CA effectively increased the ANS‐mediated fluorescence signal (Figure 4D), suggesting that covalent binding to CA significantly increased the hydrophobic exposure of ANXA5. Conversely, the increase in CA‐induced fluorescence was largely reduced in the C316A mutant protein. These findings indicate that CA caused ANXA5 protein degradation by inducing hydrophobic exposure after engaging with the C316 residue.

We explored the relationship between CA‐induced ANXA5 protein degradation and SASP secretion inhibition in senescent cells. A previous study demonstrated that ANXA5 is required for the recruitment of PKCθ to the membrane and its activation, playing a key role in the subsequent NF‐κB activation in T cells [26]. Moreover, NF‐κB signaling is the major pathway responsible for SASP production [27]. According to these findings, we speculated that CA inhibited SASP secretion by inducing ANXA5 protein degradation, causing inhibition of the PKCθ‐NF‐κB signaling cascade in senescent cells. The immunofluorescence assay indicated that 25 µm CA treatment reduced the expression of phosphorylated PKCθ as well as ANXA5 in senescent A549 cells (Figure 4E). Furthermore, a Western blot confirmed the status of the above proteins in CA‐treated senescent and non‐senescent cells. Compared to the non‐senescent cells, the protein expression levels of phosphorylated PKCθ and NF‐κB, as well as the total ANXA5, significantly increased in senescent cells (Figure 4F). CA treatment reduced ANXA5 expression as well as the activated PKCθ and NF‐κB levels (Figure 4F). Finally, in ANXA5‐knocked down senescent A549 cells, CA could not effectively regulate the activation of PKCθ and NF‐κB (Figure 4G), indicating that the effect of CA relied on ANXA5. Overall, our results suggest that CA induced ANXA5 protein degradation to inhibit the PKCθ‐NF‐κB signaling cascade in senescent cells (Figure 4H).

### 2.5 CA Alleviated Lung Fibrosis in Bleomycin‐Induced Mice Model

**FIGURE 5:**
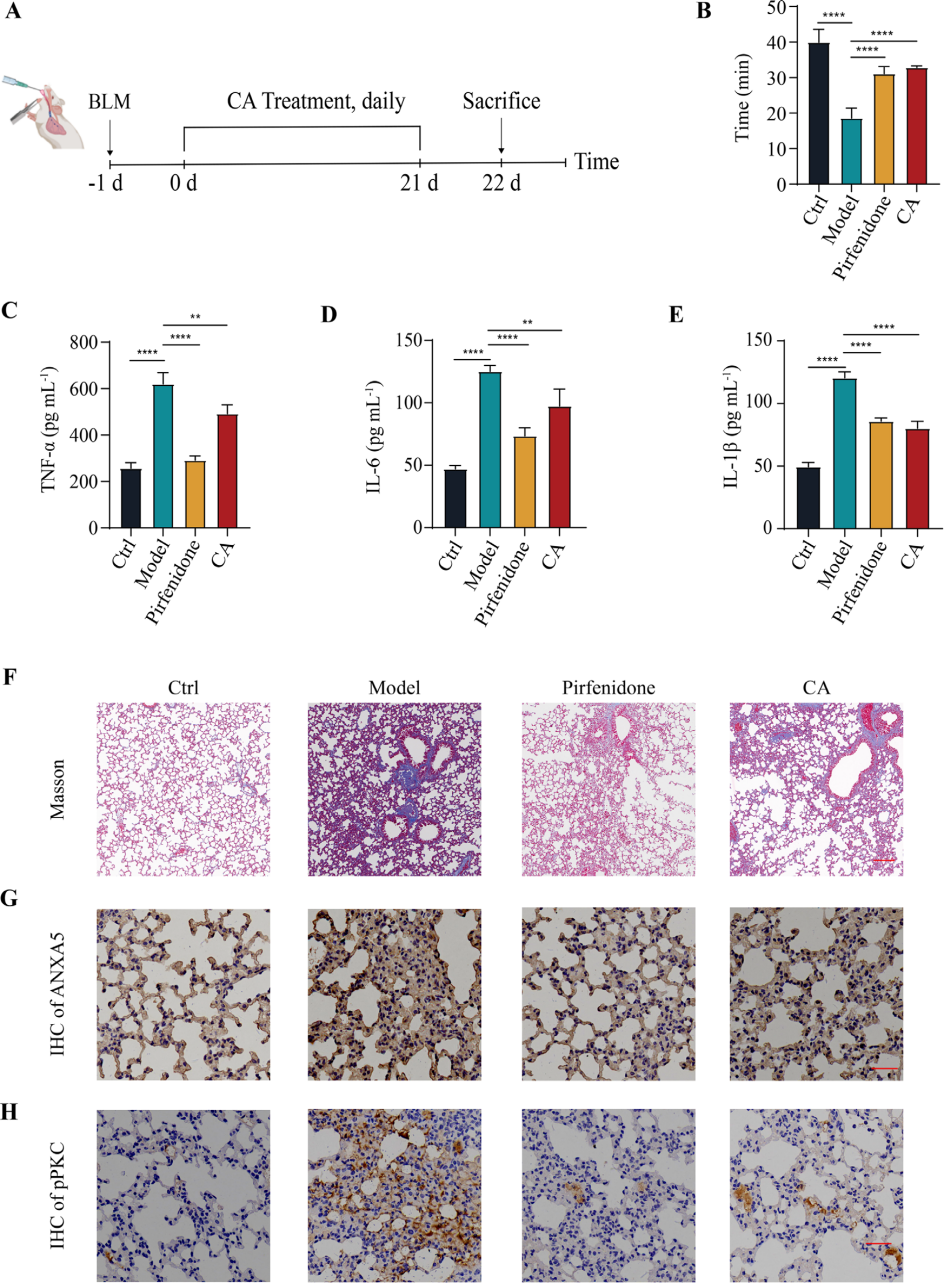
CA alleviated lung fibrosis in BLM‐induced mice model. (A) Experimental design for assessing the effect of CA in BLM‐induced pulmonary fibrosis mouse models. (B) CA increased the physical function of BLM‐treated mice in treadmill assay. Ctrl: wild‐type mice were used as the control group (*n* = 5). Model: BLM‐induced mice treated with saline as the model group (*n* = 5). Pirfenidone: BLM‐induced mice treated with 100 mg kg^−1^ Pirfenidone by gavage were the positive group (*n* = 5). CA: BLM‐induced mice treated with 10 mg kg^−1^ CA by gavage (*n* = 5). (C–E) CA decreased the levels of TNF‐α (C), IL‐6 (D), and IL‐1β (E) in the serum of model mice, *n* = 5. (F) Representative Masson staining of lungs from model mice after CA or pirfenidone treatment. Scale bar, 100 µm. (G) CA reduced the protein level of ANXA5 in the lungs of model mice as detected by immunohistochemistry assay. Scale bar, 100 µm. (H) CA decreased the protein level of the phosphorylated form of PKCθ in the lungs of model mice via immunohistochemistry assay. Scale bar, 100 µm. Data are the mean ± s.e.m. For (*B*‐*E*), a one‐way ANOVA test was used. *P < 0.05, **P < 0.01, ***P < 0.001, ****P < 0.0001. ns indicates no significance.

Having confirmed the senomorphic activity of CA and its mechanism of action in cellular models, we examined its effectiveness in an animal model. The bleomycin (BLM)‐induced lung fibrosis mouse model was utilized because of the accumulation of senescent cells in the lung as well as its widespread use for the regulation of senescent cells [4, 28]. The protective effect of CA on mouse lung injury was examined in a prophylactic mode, as depicted in Figure 5A. On the second day after intratracheal instillation of BLM in mice, 10 mg kg^−1^ of CA was administered daily via gavage for three consecutive weeks, whereas the FDA‐approved drug Pirfenidone (100 mg kg^−1^, i.g. daily) was employed as the positive control. A treadmill assay was performed to assess the physical function of mice in all groups at the conclusion. Compared to the model mice, the exercise time of mice in the CA‐treated group was extended by nearly 10 min, mirroring the positive control group (Figure 5B). We detected the levels of multiple inflammatory factors in mouse serum by ELISA and found that CA treatment decreased the concentrations of TNF‐α, IL‐6, and IL‐1β (Figure 5C–E), consistent with the cellular model.

We dissected the pathological sections of mouse lungs to validate the molecular mechanism of CA in vivo. The hematoxylin and eosin (HE) staining results indicated that the pulmonary pathological features, like thickening of the pulmonary septum, were significantly ameliorated in CA‐treated mice (Figure S7). Critically, we observed a significant decrease in fibrosis levels in the lungs of model mice following CA treatment, supported by the Masson staining results (Figure 5F). We also found the decreased expression of the pulmonary fibrosis‐related gene *Col1a1* and reduced protein levels of COL1A1 and TGF‐β in CA‐treated lung tissues (Figures S8 and S9). As we have confirmed that CA could induce ANXA5 degradation and inhibit PKCθ activation at the cellular level, we next validated this finding in mouse models. The immunohistochemistry data strongly indicated that the expression of ANXA5 protein in the lungs of CA‐treated mice was significantly reduced compared to the model group (Figure 5G). Correspondingly, the phosphorylated form of PKCθ also decreased following CA treatment (Figure 5H). In conclusion, our results indicate that CA alleviated lung fibrosis by targeting the ANXA5‐PKCθ‐NF‐κB signaling axis in BLM‐induced mouse models.

### 2.6 CA Reduced SASP Secretion and Inflammation Levels in the Lung Tissues

**FIGURE 6:**
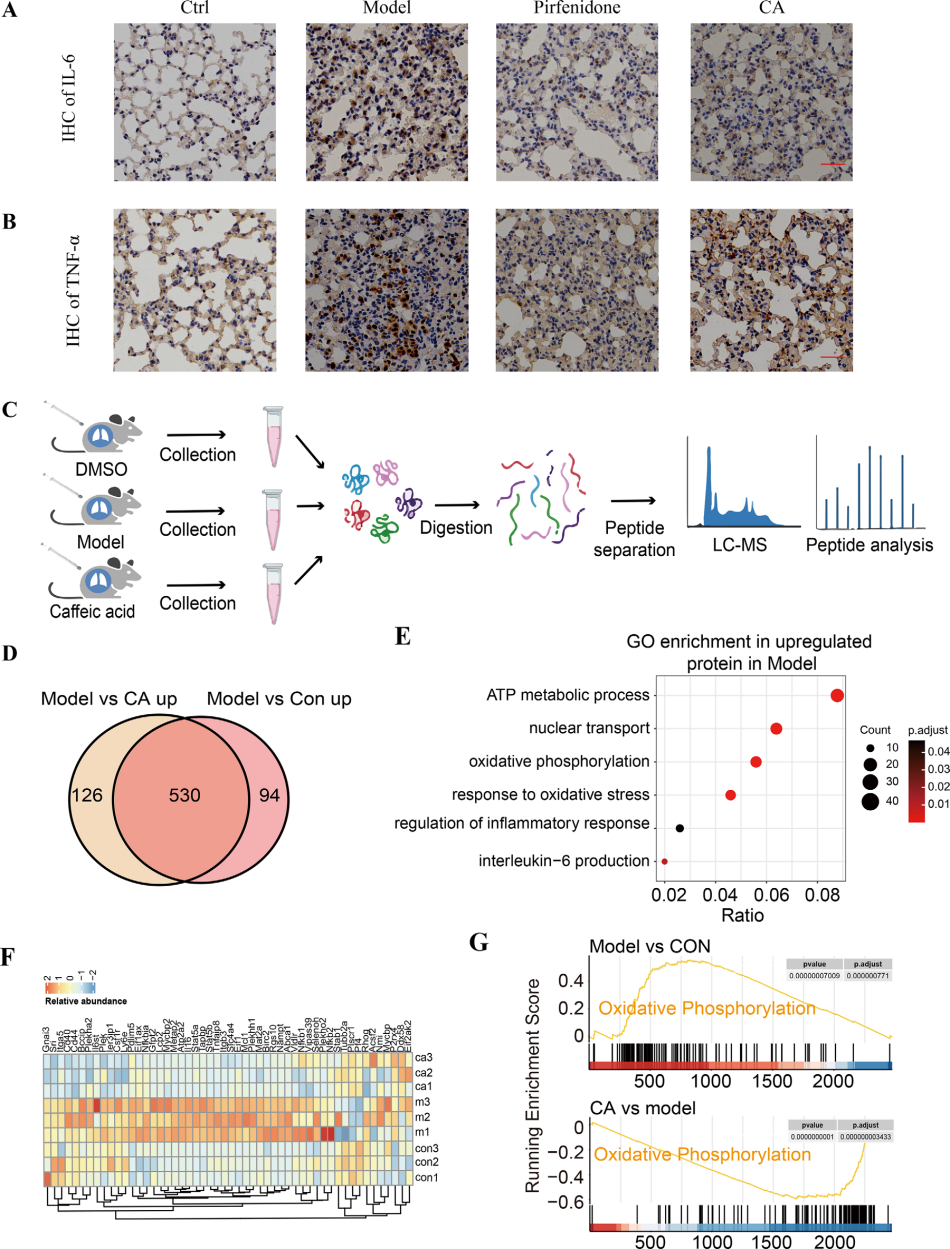
CA reduced SASP secretion and inflammation levels in lung tissues. (A, B) CA reduced the protein level of IL‐6 (A) and TNF‐α (B) in the lungs of model mice via immunohistochemistry. Scale bar, 100 µm. (C) Schematic diagram of proteomics analysis of lungs from control, model, and CA‐treated mice. (D) Venn diagram depiction of CA‐mediated protein expression changes in mouse lungs. (E) GO pathway analysis of upregulated proteins via CA treatment. (F) CA reduced the expression of inflammation‐related proteins in the lungs of model mice, as depicted by the heatmap. (G) Gene set enrichment analysis (GSEA) depicting the changes in gene signatures linked to oxidative phosphorylation following CA treatment.

We tested whether the SASP secretion in the mouse lungs was influenced by CA treatment. The expression of IL‐6 and TNF‐α was characterized by immunohistochemistry, and the results indicated that the levels of the two typical inflammatory factors were significantly suppressed in the CA‐treated group compared to the model mice (Figure 6A,B). To analyze the changes in pulmonary inflammatory levels, we performed proteomic analyses using lung tissue samples, as presented in Figure 6C. The results indicated that 624 proteins were downregulated in the lungs of the model mice compared to the control group, while 656 proteins were upregulated following CA treatment (Figure 6D). Notably, 530 proteins overlapped between the two groups of changed proteins, suggesting the effective regulatory activity of CA at the global proteomic level (Figure 6D). Gene ontology (GO) analysis revealed that these regulated proteins are associated with many important physiological processes, including metabolism, oxidative phosphorylation, inflammatory response, and IL‐6 production (Figure 6E). Aligned with this data, the expression of inflammation‐related proteins was significantly decreased in the CA‐treated group, as depicted in the heatmap of the proteomics data (Figure 6F). Additionally, we conducted gene set enrichment analysis (GSEA) and found that CA treatment in lung tissues from model mice could downregulate the gene signatures associated with oxidative phosphorylation (Figure 6G). We conducted immunofluorescence assays and found that CA did not affect the accumulation of p16‐positive senescent cells in lung slices, but the expression of IL‐6 in these cells clearly decreased (Figure S10). These results demonstrated that CA effectively reduced the SASP secretion and inflammation levels in the lung tissues of model mice.

## 3 Discussion

Healthy nutritional supplements can delay individual aging and reduce the occurrence of age‐related diseases [29]. Nutritional senomorphics are derived from food sources, such as resveratrol, kaempferol, apigenin, and epigallocatechin gallate (EGCG) [30]. Although these reported nutritional senomorphics can exhibit certain inhibitory activities on SASP secretion in senescent cells as well as in aged mouse models [31], there is a lack of defined protein target analysis and in‐depth mechanism research, particularly related to pulmonary fibrosis. In our screening system presented, we found that caffeic acid exhibited the best activity to inhibit SASP secretion in different types of senescent cells among tested molecules (Figure 1). Furthermore, using a series of chemical biology approaches including competitive ABPP technology, we successfully identified the ANXA5 protein as a direct protein target of CA (Figure 2). Compared to previously reported senomorphics, we clearly identified the cellular target and mechanism of action of CA, and demonstrated the satisfactory effects of CA in alleviating pulmonary fibrosis in mouse models as a prophylactic. Consistent with our findings, other research groups have demonstrated that the derivative of CA, caffeic acid phenethyl ester (CAPE), could attenuate lung fibrosis and pulmonary damage in BLM‐induced rats [32, 33, 34]. Caffeic acid is a phenolic acid compound commonly derived from different sources such as fruit, vegetables, tea, coffee, and herbal medicine [15, 16]. As an antioxidant, CA's antioxidant effect might play a role in mitigating inflammatory responses in senescent cells and mouse lungs. Therefore, CA is a promising senomorphic with the potential to be used as a nutritional factor to prevent pulmonary fibrosis.

Importantly, ANXA5 has also been shown to be an attractive protein target for promising senomorphics to regulate senescent cells. We have demonstrated that ANXA5 protein degradation mediated by hydrophobic exposure inhibits the activation of PKCθ and NF‐κB, causing reduced SASP secretion (Figure 3). Previous studies have demonstrated the critical role of ANXA5 in PKCθ translation and NF‐κB signaling in the T cell activation process [26]. Integrated with our experimental evidence, it can be concluded that this ANXA5‐PKCθ‐NF‐κB signaling pathway is relevant in senescent cells. Considering the primary regulatory role of the NF‐κB pathway in SASP secretion [27], targeting upstream of ANXA5 would inhibit the release of inflammatory factors mediated by downstream NF‐κB. Interestingly, ANXA5 is a biomarker for cellular aging [22]. Increased alveolar soluble ANXA5 was observed in human bronchoalveolar lavage fluids (BALF) with interstitial lung disease, and its expression significantly increased in serum and lung tissues in silica‐exposed mice [35, 36]. Consistent with these results, we found that the ANXA5 protein level was increased in senescent cells and fibrotic mouse lungs (Figures 4F,G and 5G). Due to the crucial role of senescent cells in age‐related disease pathogenesis, ANXA5 could be used as an effective and specific target for developing novel therapies for these diseases.

## 4 Conclusions

To uncover promising senomorphics with defined protein targets and mechanisms of action, we conducted screening and identified CA as a potent nutritional senomorphic. Utilizing chemical biology approaches, the ANXA5 protein was identified as a covalent protein target of caffeic acid in senescent cells. Further mechanistic research suggested that the direct binding of caffeic acid to Cys316 on ANXA5 induced its hydrophobic exposure and degradation, triggering the inhibition of PKCθ and NF‐κB activation as well as reduced SASP secretion. Moreover, CA alleviated lung fibrosis and decreased inflammation levels by targeting ANXA5 in bleomycin‐induced mouse models. Our research indicates that CA is a novel nutritional senomorphic with promising application prospects, and Annexin A5 could be developed as a practical and specific target for precise intervention in aging‐related diseases.

## 5 Experimental Section

### 5.1 Reagents and Materials

CA (purity ≥ 98%) was obtained from Beijing Bethealth People Biomedical Technology (Beijing, China). Cisplatin (HY‐17394), Bafilomycin A1 (88899‐55‐2), and MG132 (133407‐82‐6) were acquired from MedChem Express (USA). 1‐anilino‐8‐naphthalene sulfonate (ANS, A1028) was obtained from Sigma Aldrich. Click chemistry reaction and LC‐MS/MS reagents included TBTA (1770049), TCEP (C4706), Rhodmine‐N_3_ (83689), and CuSO_4_ (C1297), which were bought from Sigma (USA). TMT 10plex reagent (A34808), high‐capacity neutravidin agarose resin (A53031), and trypsin (90057) were obtained from Thermo Fisher (USA). Additionally, specific primary antibodies against Annexin A5 (11060‐1‐AP), phospho‐NF‐κB (82335‐1‐RR), NF‐κB (80979‐1‐RR), IL‐6 (21865‐1‐AP), p21 (10355‐1‐AP), p16 (10883‐1‐AP), and TNF‐α (60291‐1‐Ig) were purchased from Proteintech (Wuhan, China). The specific primary antibodies against phospho‐PKCθ (sc‐271922) and PKCθ (sc‐1680) were acquired from Santa Cruz Biotechnology (Shanghai, China). The specific primary antibodies against β‐actin (GB15001), Tubulin (GB15140), COL1A1 (GB11022‐3‐100) and TGF‐β (GB11179‐100) were purchased from Saiweier Biotechnology (Wuhan, China).

### 5.2 Cell Culture

A549 and BEAS‐2B cells were acquired from the Chinese Academy of Medical Sciences (Beijing, China). A549 and BEAS‐2B cells were maintained at 37°C and 5% CO_2_ in DMEM (Corning, USA) supplemented with 10% FBS (Corning, USA) and 100 IU penicillin‐streptomycin (Thermo Fisher, USA). The senescent A549 cells were induced using 15 µm cisplatin treatment for 72 h. BEAS‐2B cells were exposed to UVA radiation for 20 min to induce senescence. The senescent cells were validated through SA‐β‐gal staining.

### 5.3 Screening and Confirming of Senomorphics

To screen effective senomorphics, we performed the following experiments. Initially, we seeded senescent and non‐senescent A549 cells at 1 × 10^5^ cells per well in a 24‐well plate. These cells were incubated with 30 different natural compounds at a concentration of 10 µm for 24 h. We assessed the levels of inflammatory factors in the supernatant using ELISA. We characterized compounds that could effectively reduce the levels of inflammatory factors as senomorphics.

To verify the activity of the identified senomorphics, senescent and non‐senescent cells (A549 and BEAS‐2B) were cultured in a 6‐well culture plate at 1×10^6^ cells/well for 24 h. Cells were exposed to different concentrations of CA (0, 3, 6, 12, 25 µm and 0, 25, 50, 100 µm) for another 24 h. The levels of inflammatory factors in the cell supernatant were measured using ELISA.

### 5.4 SA‐β‐Gal Staining

The SA‐β‐gal staining kit (C0602, Beyotime Biotechnology, Shanghai) was used in this study. Cells were fixed for 15 min. Next, the β‐gal staining solution was applied and incubated overnight at 37°C. Finally, the cells were rinsed with PBS and imaged using a light microscope.

### 5.5 Competitive in‐gel Fluorescence Labeling Assay

Senescent A549 cells (1 × 10^6^ cells/well) were plated onto a 6 cm plate and treated with different concentrations of CA (0, 20, 40, and 80 µm) for 2 h. After harvesting the cells, 100 µL of RIPA buffer was employed for cell lysis. The protein concentration was assessed, and the cells were labeled with a cysteine‐specific probe (IAA‐yne) for 1 h at 37°C on a shaker. A buffer containing 9 µL of TBTA (10 mm in DMSO), 3 µL of TCEP (50 mm in ddH_2_O), 3 µL of CuSO_4_ (50 mm in ddH_2_O), and 1 µL of TAMRA azide compound (10 mm in DMSO) was added to each sample and incubated for 2 h at 37°C on a shaker. After that, 1 mL of acetone was added to precipitate the labeled protein. The precipitated protein was centrifuged at 20 000 × *g* for 10 min at −80°C, and the supernatant was removed. The residual acetone was evaporated, and the sample was dissolved in 100 µL of 1× loading buffer solution and denatured by heating at 90°C for 5 min. The denatured sample was separated using a 12% SDS‐PAGE gel, and the fluorescence was scanned using an Azure Sapphire RGB NIR scanner. The total proteins were visualized using CBB staining.

### 5.6 Pull‐Down Assay

Pull‐down assays were performed to validate protein interactions with CA. The samples were separated into three groups: DMSO, IAA‐yne, and CA+ IAA‐yne, as described earlier. Subsequently, a click chemical reaction was performed using 9 µL of 10 mm TBTA, 3 µL of 50 mm TCEP, 3 µL of 50 mm CuSO_4_, and 1 µL of 5 mm TAMRA‐N3 in a 100 µL mixture for 2 h. The labeled protein was precipitated using acetone, dissolved in 1 mL of 1.2% SDS/PBS, and heated at 95°C for 15 min. The soluble proteins were diluted to 0.1% SDS with 1×PBS and centrifuged at 12 000 × *g* for 10 min at room temperature. The beads were harvested through centrifugation at 1400 × *g* for 3 min, and rinsed sequentially with 1% SDS, 0.1% SDS, and 6 m urea. Finally, the proteins separated and enriched by SDS‐PAGE gel were immunoblotted after adding 1×loading buffer solution and denaturing at 90°C for 10 min. Western blotting was performed using the anti‐Annexin A5 antibody.

### 5.7 Streamlined Cysteine Activity‐based Protein Profiling

We employed competitive mass spectrometry experiments to characterize potential protein targets of CA in senescent cells. First, we treated the senescent A549 cells with DSMO, 40 or 80 µm caffeic acid for 2 h, and lysed the cells before adding 100 µm DBIA probe (Chomix Biotech Co, Nanjing, China) for labeling in a shaker at 37°C. Next, we alkylated the reduced cysteine residues by adding 5 mm DTT and 20 mm IAA, allowing them to react in the dark for 30 min. The protein was digested by adding 2 µg of trypsin. Subsequently, we performed peptide labeling and enrichment using TMT10plex Mass Tag reagent (Thermo Scientific, USA). To enrich the peptides, we added 100 µL Streptavidin beads (Thermo Scientific, USA) to the TMT‐labeled mixed samples dissolved in 1 mL of PBS. To remove non‐specific binding, we rinsed the beads sequentially with 1 mL PBS, 1 mL 0.1% SDS, and 1 mL ddH_2_O 3 times. Finally, we desalted the peptide using 0.1% formic acid and a commercial C18 column before subjecting it to mass spectrometry.

### 5.8 Competitive In‐Gel Fluorescence Labeling of ANXA5 Protein

Tyrosine was incubated with CA to generate the oxidized form of CA. For labeling competition experiments, the ANXA5 protein was pretreated with CA, oxidized CA, and IAA for 1 h. After that, it was labeled using an IAA‐yne probe. The chemical reaction was then clicked to proceed under the above conditions. Ultimately, the samples were separated using a 12% SDS‐PAGE gel, and fluorescence was scanned. The total proteins were observed using CBB staining.

### 5.9 Caffeic Acid Probe Synthesis


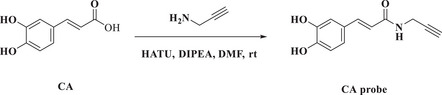


Caffeic acid (0.2 mmol, 36 mg) and 1‐amino‐2‐propargyl (0.4 mmol, 22 mg) in anhydrous *N*,*N*‐dimethylformamide (DMF, 2 mL), 2‐(7‐azabenzotriazol‐1‐yl)‐*N*,*N*,*N*',*N*'‐tetramethyluronium hexafluorophosphate (HATU, 0.3 mmol, 114 mg), and *N*,*N*‐diisopropylethylamine (DIPEA, 0.6 mmol, 77.4 mg) catalyst were added. The solution was stirred at ambient temperature overnight via magnetic stirring. After the reaction concluded, water (1 mL) and ethyl acetate (10 mL) were added to the solution. The organic phase was separated from the aqueous phase, dried with anhydrous NaSO_4_, and concentrated. The residue was purified by column chromatography (dichloromethane/methanol = 8:1) to harvest the caffeic acid probe.

^1^H NMR (400 MHz, MeOD) *δ* 7.32 (*d*, *J* = 15.7 Hz, 1H), 6.91 (*d*, *J* = 2.0 Hz, 1H), 6.83–6.79 (*m*, 1H), 6.67 (*d*, *J* = 8.2 Hz, 1H), 6.25 (*d*, *J* = 15.7 Hz, 1H), 3.96 (*d*, *J* = 2.5 Hz, 2H), 2.50 (*t*, *J* = 2.6 Hz, 1H). ^13^C NMR (126 MHz, MeOD‐D6) *δ*167.44, 147.59, 145.34, 141.50, 126.75, 120.83, 116.31, 115.05, 113.70, 79.20, 70.81, 28.13. High‐resolution mass spectrometry (HRMS) *m*/*z*: [M+H]^+^ calculated for C_12_H_11_O_3_N 218.0817 and found 218.0817.

### 5.10 In‐Gel Fluorescence Labeling of Wild‐Type and Mutant ANXA5 Protein Using the CA Probe

We constructed wild‐type and C316A mutant ANXA5 plasmids and purified recombinant proteins from *E*. *coli*. Then, we used the CA probe to label the wild‐type and mutant ANXA5 protein, followed by a click chemistry reaction as described above. Finally, the samples were separated on a 12% SDS‐PAGE gel and subjected to fluorescence scanning. Total proteins were observed via CBB staining.

### 5.11 Cellular Imaging

Fluorescence microscopy was used to determine the co‐localization of ANXA5 and IAA‐yne, as well as pPKCθ, in senescent A549 cells (5×10^4^ cells/well) inoculated into 4‐chamber glass plates (Cellvis) following CA treatment. For the co‐localization of ANXA5 and pPKCθ, the senescent A549 cells were separated into two groups: DMSO‐treated and 25 µm CA‐treated. To prepare the cells for imaging, they were fixed with cell fixative for 20 min, soaked in 0.2% Triton X‐100 for 15 min, and mixed with anti‐ANXA5 antibody (1:500) and anti‐pPKCθ antibody (1:1000) overnight at 4°C. The next day, the cells were incubated with goat anti‐rabbit IgG (1:1000, Alexa Fluor 488) for ANXA5 and with goat anti‐rabbit IgG (1:1000, Alexa Fluor 488) for pPKCθ for 2 h. The cells were observed using a laser scanning confocal fluorescence microscope (Leica TCS SP8).

### 5.12 Microscale Thermophoresis Assay (MST)

The *K*_D_ value between CA and ANXA5 protein was determined using microscale thermophoresis (NT.115, Nano Temper Technologies). CA solutions of 1 mm were diluted and mixed with ANXA5 protein solutions (0.2 mg mL^−1^) labeled using a Monolith His‐Tag Labeling Kit (MO‐L018, NanoTemper Technologies) with equal volumes. These mixtures were placed into separate capillaries and analyzed using MST with 80% power. The direct binding of ANXA5 to CA was identified by measuring the difference in thermophoresis of the fluorescently labeled protein upon complex formation. The data was analyzed using MO Affinity Analysis Software (v2.3).

### 5.13 Cellular Thermal Shift Assay (CETSA)

Senescent A549 cell lysis was incubated with DMSO or 50 µm CA for 2 h at room temperature, and heated over a gradient of 37–67°C for 3 min. The cell lysis was clarified by centrifugation at 15,000 rpm (ST16R, ThermoFisher Scientific) at 4°C for 15 min. The supernatant was analyzed via immunoblotting.

### 5.14 Molecular Docking

The 3D structure of ANXA5 (PDB ID: 1ANW) was acquired from the RCSB Protein Data Bank (https://www.rcsb.org/). The ANXA5 protein and CA chemical structure were processed using Autodock software (version 4.2) to hydrogenate and dehydrate structures. Molecular docking was performed using Discovery Studio 2019 client (version 2.5) to define the optimized binding conformation of the ANXA5‐CA system. Intermolecular interactions were assessed using Pymol software (version 2.5) for mapping and analysis.

### 5.15 RNA Interference

The siRNA for ANXA5 (5'‐GACAAGUACAUGACUAUAU‐3') and its negative control (5'‐UUCUCCGAACGUGUCACGU‐3') were purchased from Jima Pharmaceutical Company (Suzhou, China). We transfected A549 cells with 20 nm Lipofectamine 2,000 to knock out ANXA5 following the instructions (Thermo Fisher). To verify whether siRNA successfully knocked out ANXA5, we performed a Western blot analysis. The levels of inflammatory factors in the supernatants of cells transfected with scrambled siRNA or ANXA5 siRNA were determined by ELISA.

### 5.16 Western Blotting

Proteins from 1 × 10^6^ senescent and non‐senescent A549 cells were acquired by lysing the cells using lysis buffer (P0013K and P1112, Beyotime). The proteins were separated using SDS‐PAGE and transferred to PVDF membranes. The PVDF membranes were incubated with primary and secondary antibodies, and the resulting protein bands were visualized with the Azure C400 system and analyzed using Image J software.

### 5.17 UV Spectroscopic Measurements for Protein Structure Changes

The structural changes of the wild‐type and C311A mutant ANXA5 protein before and after CA treatment were characterized using Bis‐ANS (b153, Thermo Scientific, USA), a fluorescent probe for non‐polar cavities in proteins. CA (100 µM) was added to PBS buffer solutions containing 5 µg wild‐type or C316A mutant ANXA5 protein. Then, full‐wavelength scans were conducted over 400 to 700 nm. The scanning conditions were recorded using a microplate reader (PerkinElmer Envision, UK).

### 5.18 Pulmonary Fibrosis Mouse Model

Male BALB/c mice aged 5–8 weeks were obtained from the Beijing Vital River Laboratory Animal Technology Company. Except for the control group with the wild‐type mice, the other mice were given an endotracheal intubation of 5 mg kg^−1^ BLM to induce pulmonary fibrosis. The BLM‐treated BALB/c mice were randomly divided into three groups. The CA group, the pirfenidone group, and the model group were administered 10 mg kg^−1^ CA, 100 mg kg^−1^ pirfenidone, and saline daily via gavage for 3 weeks. The mice's body weight was measured throughout the experiment. Then, 3 weeks prior to the treadmill test, all mice were euthanized. The internal organs of each group were collected and fixed in 4% paraformaldehyde for Masson staining, HE staining, IHC, and characterization of inflammatory factors in lung tissue. All animal experiments were performed in compliance with the institutional ethics committee regulations and guidelines on animal welfare with the approval of the China Academy of Chinese Medical Sciences (approval number: 2023B298).

### 5.19 Detection of the Expression of *Col1a1*

Total RNA was extracted from lung tissues of each group using Trizol reagent, and the concentration of RNA was determined. RNA was reverse transcribed into cDNA, and cDNA was utilized as a template for qPCR amplification. A real‐time PCR detection system equipped with a thermal cycler and an optical detection module measured the fluorescence signal produced at each amplification cycle. The relative expression of the gene was calculated using 2^−∆∆Ct^. All primers used for PCR were synthesized by Shanghai Sangong Biological Company (Shanghai, China), and the primer sequences are presented in Table 1.

**TABLE 1:** Primer sequence.

Primer	Sequence (5′–3′)
Col1a1 forward	TGCCGTGACCTCAAGATGTG
Col1a1 reverse	CACAAGCGTGCTGTAGGTGA
GAPDH forward	CTTTGTCAAGCTCATTTCCTGG
GAPDH reverse	TCTTGCTCAGTGTCCTTGC

### 5.20 Whole‐Proteome Experiments

Lung tissues obtained from control, BLM‐induced, and CA groups were lysed. Next, the protein concentrations were determined using a BCA kit. After that, 5 mm DTT and 10 mm IAA were added and incubated for 30 min in a 37°C shaker. Then, trypsin digestion was performed overnight, followed by commercial C18 column desalination before detection. Finally, the prepared samples were subjected to LC‐MS/MS.

### 5.21 GO and KEGG Enrichment Analysis

The abundance changes of the three groups across the whole proteome were used to identify differential proteins based on absolute fold change ≥ 1.5 and *P*‐value (FDR) < 0.05. We constructed Volcano maps using the bioladder website (https://www.bioladder.cn). Biological process and KEGG pathway enrichment were selected to visualize functional profiles after analyzing the differential proteins by DAVID+ (https://david.ncifcrf.gov/).

### 5.22 Statistical Analysis

GraphPad Prism 8.0 software (GraphPad Prism, USA) was utilized to perform Statistical analysis. All data are presented as mean ± s.e.m, and evaluated with Student's *t*‐test, one‐way or two‐way ANOVA test. Differences were considered statistically significant at *P* < 0.05, < 0.01, < 0.001, or < 0.0001.

## Author Contributions

Yinhua Zhu, Ying Zhang, Qianyu Zhang, and Ping Song designed the research, performed the majority of experiments, and wrote the manuscript. Junzhe Zhang, Ang Ma, and Chen Wang performed the omics data analysis. Peng Gao, Tong Yang, and Lirun Zhou helped conduct the animal experiments. Qiaoli Shi and Yin Kwan Wong helped to revise the manuscript. Yongting Luo, Huan Tang, and Jigang Wang designed, supervised the research, and wrote the manuscript.

## Conflicts of Interest

The authors declare no conflicts of interest.

## Supporting information




**Supporting File 1**: exp270099‐sup‐0001‐SuppMat.docx

## Data Availability

The data that support the findings of this study are available from the corresponding author upon reasonable request.

## References

[1] F. J. Martinez , H. R. Collard , A. Pardo , et al., “Idiopathic Pulmonary Fibrosis,” Nature Reviews Disease primers 3 (2017): 17074. 10.1038/nrdp.2017.74 29052582

[2] T. M. Maher , “Interstitial Lung Disease,” JAMA 331 (2024): 1655. 38648021 10.1001/jama.2024.3669

[3] D. J. Lederer and F. J. Martinez , “Idiopathic Pulmonary Fibrosis,” New England Journal of Medicine 378 (2018): 1811–1823. 29742380 10.1056/NEJMra1705751

[4] M. J. Schafer , T. A. White , K. Iijima , et al., “Cellular Senescence Mediates Fibrotic Pulmonary Disease,” Nature Communications 8 (2017): 14532. 10.1038/ncomms14532 PMC5331226 28230051

[5] C. Yao , X. Guan , G. Carraro , et al., “Senescence of Alveolar Type 2 Cells Drives Progressive Pulmonary Fibrosis,” American Journal of Respiratory and Critical Care Medicine 203 (2021): 707–717. 32991815 10.1164/rccm.202004-1274OC PMC7958503

[6] P. J. Barnes , J. Baker , and L. E. Donnelly , “Cellular Senescence as a Mechanism and Target in Chronic Lung Diseases,” American Journal of Respiratory and Critical Care Medicine 200 (2019): 556–564. 30860857 10.1164/rccm.201810-1975TR

[7] J. Chang , Y. Wang , L. Shao , et al., “Clearance of Senescent Cells by ABT263 Rejuvenates Aged Hematopoietic Stem Cells in Mice,” Nature Medicine 22 (2016): 78–83. 10.1038/nm.4010 PMC4762215 26657143

[8] L. J. Niedernhofer and P. D. Robbins , “Senotherapeutics for Healthy Ageing,” Nature Reviews Drug Discovery 17 (2018): 377–377. 10.1038/nrd.2018.44 29651106

[9] Y. Zhu , T. Tchkonia , H. Fuhrmann‐Stroissnigg , et al., “Identification of a Novel Senolytic Agent, Navitoclax, Targeting the BCL‐2 Family of Anti‐Apoptotic Factors,” Aging Cell 15 (2016): 428–435. 26711051 10.1111/acel.12445 PMC4854923

[10] Y. Zhu , T. Tchkonia , T. Pirtskhalava , et al., “The Achilles' Heel of Senescent Cells: From Transcriptome to Senolytic Drugs,” Aging Cell 14 (2015): 644–658. 25754370 10.1111/acel.12344 PMC4531078

[11] J. C. Cooley , N. Javkhlan , J. A. Wilson , et al., “Inhibition of Antiapoptotic BCL‐2 Proteins With ABT‐263 Induces Fibroblast Apoptosis, Reversing Persistent Pulmonary Fibrosis,” JCI Insight 8 (2023): e163762. 36752201 10.1172/jci.insight.163762 PMC9977433

[12] J. N. Justice , A. M. Nambiar , T. Tchkonia , et al., “Senolytics in Idiopathic Pulmonary Fibrosis: Results From a First‐in‐Human, Open‐Label, Pilot Study,” EBioMedicine 40 (2019): 554–563. 30616998 10.1016/j.ebiom.2018.12.052 PMC6412088

[13] A. Nambiar , D. Kellogg , J. Justice , et al., “Senolytics Dasatinib and Quercetin in Idiopathic Pulmonary Fibrosis: Results of a Phase I, Single‐Blind, Single‐Center, Randomized, Placebo‐Controlled Pilot Trial on Feasibility and Tolerability,” EBioMedicine 90 (2023): 104481. 36857968 10.1016/j.ebiom.2023.104481 PMC10006434

[14] Y. Zhang , Q. Zhang , Z. Chu , et al., “Oridonin Acts as a Novel Senolytic by Targeting Glutathione S‐Transferases to Activate the ROS‐p38 Signaling Axis in Senescent Cells,” Chemical Communications (Cambridge, England) 58 (2022): 13250–13253. 36367053 10.1039/d2cc05278d

[15] S. Mirzaei , M. H. Gholami , A. Zabolian , et al., “Caffeic Acid and Its Derivatives as Potential Modulators of Oncogenic Molecular Pathways: New Hope in the Fight Against Cancer,” Pharmacological Research 171 (2021): 105759. 34245864 10.1016/j.phrs.2021.105759

[16] N. N. Muhammad Abdul Kadar , F. Ahmad , S. L. Teoh , and M. F. Yahaya , “Caffeic Acid on Metabolic Syndrome: A Review,” Molecules (Basel, Switzerland) 26 (2021): 5490. 34576959 10.3390/molecules26185490 PMC8465857

[17] K. M. M. Espíndola , R. G. Ferreira , L. E. M. Narvaez , et al., “Chemical and Pharmacological Aspects of Caffeic Acid and Its Activity in Hepatocarcinoma,” Frontiers in Oncology 9 (2019): 541. 31293975 10.3389/fonc.2019.00541 PMC6598430

[18] Y. Zhu , Y. Zhang , Q. Zhang , et al., “Gambogic Acid Suppresses the Pentose Phosphate Pathway by Covalently Inhibiting 6PGD Protein in Cancer Cells,” Chemical Communications (Cambridge, England) 58 (2022): 9030–9033. 35876000 10.1039/d2cc03069a

[19] M. Kuljanin , D. C. Mitchell , D. K. Schweppe , et al., “Reimagining High‐Throughput Profiling of Reactive Cysteines for Cell‐Based Screening of Large Electrophile Libraries,” Nature Biotechnology 39 (2021): 630–641. 10.1038/s41587-020-00778-3 PMC8316984 33398154

[20] Y. Zhu , L. Wang , J. Li , et al., “Photoaffinity Labeling Coupled With Proteomics Identify PDI‐ADAM17 Module is Targeted by (−)‐Vinigrol to Induce TNFR1 Shedding and Ameliorate Rheumatoid Arthritis in Mice,” Cell Chemical Biology 31 (2024): 452–464.e10. 37913771 10.1016/j.chembiol.2023.10.003

[21] T. H. Kang , J. H. Park , A. Yang , et al., “Annexin A5 as an Immune Checkpoint Inhibitor and Tumor‐Homing Molecule for Cancer Treatment,” Nature Communications 11 (2020): 1137. 10.1038/s41467-020-14821-z PMC7048819 32111835

[22] K. Klement , C. Melle , U. Murzik , S. Diekmann , J. Norgauer , and P. Hemmerich , “Accumulation of Annexin A5 at the Nuclear Envelope is a Biomarker of Cellular Aging,” Mechanisms of Ageing and Development 133 (2012): 508–522. 22728018 10.1016/j.mad.2012.06.003

[23] A. K. Hurben , L. N. Erber , N. Y. Tretyakova , and T. M. Doran , “Proteome‐Wide Profiling of Cellular Targets Modified by Dopamine Metabolites Using a Bio‐Orthogonally Functionalized Catecholamine,” Acs Chemical Biology 16 (2021): 2581–2594. 34726906 10.1021/acschembio.1c00629 PMC9872492

[24] I. Dikic , “Proteasomal and Autophagic Degradation Systems,” Annual Review of Biochemistry 86 (2017): 193–224. 10.1146/annurev-biochem-061516-044908 28460188

[25] G. V. Semisotnov , N. A. Rodionova , O. I. Razgulyaev , V. N. Uversky , A. F. Gripas' , and R. I. Gilmanshin , “Study of the “Molten Globule” Intermediate State in Protein Folding by a Hydrophobic Fluorescent Probe,” Biopolymers 31 (1991): 119–128. 2025683 10.1002/bip.360310111

[26] Z. Hu , L. Li , B. Zhu , et al., “Annexin A5 is Essential for PKCθ Translocation During T‐Cell Activation,” Journal of Biological Chemistry 295 (2020): 14214–14221. 32796034 10.1074/jbc.RA120.015143 PMC7549025

[27] A. Salminen , A. Kauppinen , and K. Kaarniranta , “Emerging Role of NF‐κB Signaling in the Induction of Senescence‐Associated Secretory Phenotype (SASP),” Cell Signalling 24 (2012): 835–845. 22182507 10.1016/j.cellsig.2011.12.006

[28] Y. Cai , H. Zhou , Y. Zhu , et al., “Elimination of Senescent Cells by β‐Galactosidase‐Targeted Prodrug Attenuates Inflammation and Restores Physical Function in Aged Mice,” Cell Research 30 (2020): 574–589. 32341413 10.1038/s41422-020-0314-9 PMC7184167

[29] V. D. Longo and R. M. Anderson , “Nutrition, Longevity and Disease: From Molecular Mechanisms to Interventions,” Cell 185 (2022): 1455–1470. 35487190 10.1016/j.cell.2022.04.002 PMC9089818

[30] C. Luís , A. T. Maduro , P. Pereira , J. J. Mendes , R. Soares , and R. Ramalho , “Nutritional Senolytics and Senomorphics: Implications to Immune Cells Metabolism and Aging—From Theory to Practice,” Frontiers in Nutrition 9 (2022): 958563. 36159455 10.3389/fnut.2022.958563 PMC9493043

[31] S. M. Lagoumtzi and N. Chondrogianni , “Senolytics and Senomorphics: Natural and Synthetic Therapeutics in the Treatment of Aging and Chronic Diseases,” Free Radical Biology and Medicine 171 (2021): 169–190. 33989756 10.1016/j.freeradbiomed.2021.05.003

[32] A. Larki , A. A. Hemmati , A. Arzi , M. G. Borujerdnia , S. Esmaeilzadeh , and M. R. Zad Karami , “Regulatory Effect of Caffeic Acid Phenethyl Ester on Type I Collagen and Interferon‐Gamma in Bleomycin‐Induced Pulmonary Fibrosis in Rat,” Research in Pharmaceutical Sciences 8 (2013): 243–252. 24082893 PMC3757589

[33] A. Larki‐Harchegani , A. A. Hemmati , A. Arzi , et al., “Evaluation of the Effects of Caffeic Acid Phenethyl Ester on Prostaglandin E2 and Two Key Cytokines Involved in Bleomycin‐Induced Pulmonary Fibrosis,” Iranian Journal of Basic Medical Sciences 16 (2013): 850–857. 23997916 PMC3758057

[34] H. Özyurt , S. Söğüt , Z. Yıldırım , et al., “Inhibitory Effect of Caffeic Acid Phenethyl Ester on Bleomycine‐Induced Lung Fibrosis in Rats,” Clinica Chimica Acta 339 (2004): 65–75. 10.1016/j.cccn.2003.09.015 14687895

[35] S. Buckley , W. Shi , W. Xu , et al., “Increased Alveolar Soluble Annexin V Promotes Lung Inflammation and Fibrosis,” European Respiratory Journal 46 (2015): 1417–1429. 26160872 10.1183/09031936.00002115 PMC4767328

[36] C. Luo , X. Ji , J. Fan , et al., “Annexin A5 Promotes Macrophage Activation and Contributes to Pulmonary Fibrosis Induced by Silica Particles,” Toxicology and Industrial Health 32 (2016): 1628–1638. 25757482 10.1177/0748233715572744

