# Supplementary material for: Caffeic Acid Acts as a Potent Senomorphic and Alleviates Inflammation and Lung Fibrosis by Covalently Targeting Annexin A5 Protein in Mice
Source: Exploration (Beijing). 2025 Dec 12;5(6):20240069. doi: 10.1002/EXP.20240069 (PMC12752563; doi:10.1002/EXP.20240069)
Supplement: Supplementary file 1 — Supporting File 1: exp270099‐sup‐0001‐SuppMat.docx [file EXP2-5-20240069-s001.docx]

**Caffeic acid acts as a potent** **senomorphics and** **alleviates inflammation and lung fibrosis by covalently targeting Annexin A5 protein in mice**

Yinhua Zhu ^a, #^, Ying Zhang ^b, #^, Qianyu Zhang ^b, #^, Ping Song ^b, #^, Junzhe Zhang ^b^, Ang Ma ^b^, Chen Wang ^b^, Peng Gao ^b^, Tong Yang ^b^, Lirun Zhou ^b^, Qiaoli Shi ^b^, Yin Kwan Wong ^c^, Yongting Luo ^a, *^, Huan Tang ^b, *^ and Jigang Wang ^b, *^

^a^ Beijing Advanced Innovation Center for Food Nutrition and Human Health, Department of Nutrition and Health, China Agricultural University, Beijing 100193, China

^b^ State Key Laboratory for Quality Ensurance and Sustainable Use of Dao-di Herbs, Artemisinin Research Center, and Institute of Chinese Materia Medica, China Academy of Chinese Medical Sciences, Beijing 100700, China

^c^ Department of Biological Sciences, National University of Singapore, Singapore

^*^ Corresponding authors.

E-mail addresses: jgwang@icmm.ac.cn (J. Wang), htang@icmm.ac.cn (H. Tang), luo.yongting@cau.edu.cn (Y. Luo).

^#^ These authors contributed equally to this work


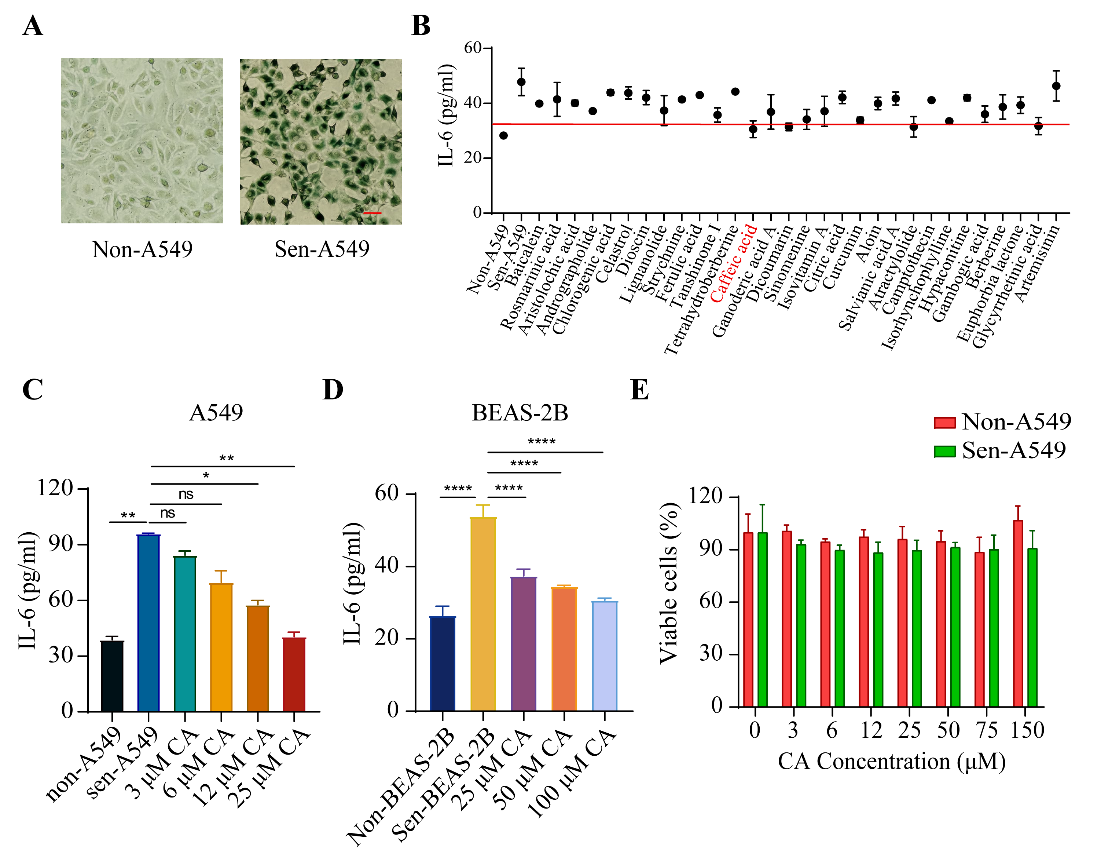


Fig. S1A. Identification of senescent cells by SA-β-gal activity assay. Scale bar, 10 μm.

Fig. S1B. Detection of the concentration of IL6 in the culture medium of senescent A549 cells treated with 10 μM compound for 24 h.

Fig. S1C and S1D CA dose-dependently decreased the concentration of IL6 in the culture medium of senescent A549 (C) and BEAS-2B (D) cells, n=3.

Fig.S1E. Assay of cell viability of senescent and non-senescent A549 cells treated with different concentrations of CA for 24 h, n=3.

Data are mean ± s.e.m. For (*C*-*D*), one-way ANOVA test used. *P<0.05, **P<0.01, ***P<0.001, ****P<0.0001. ns means no significance.

^
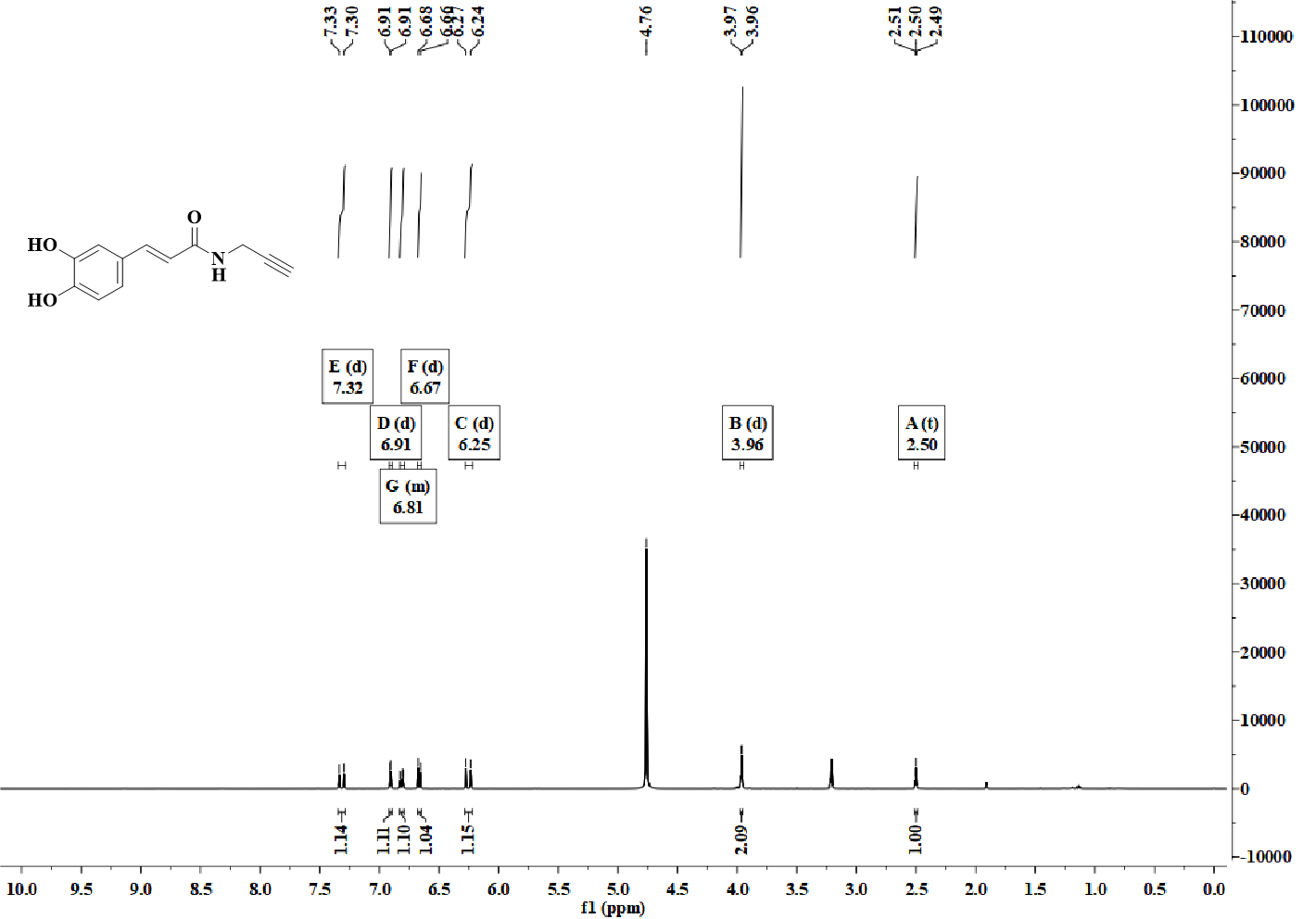
^

Fig.S2. ^1^H NMR spectrum of caffeic acid probe

^
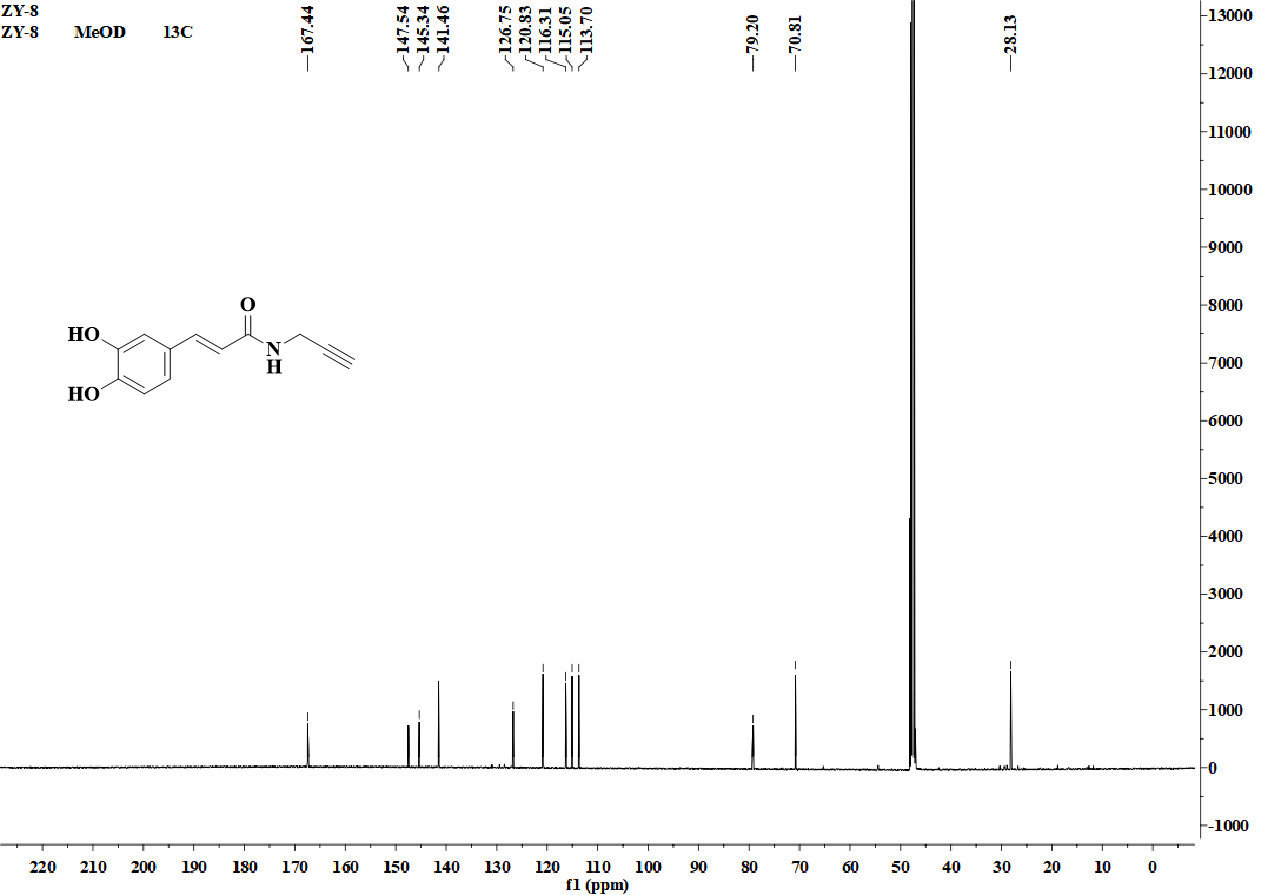
^

Fig. S3. ^13^C NMR spectrum of caffeic acid probe

^
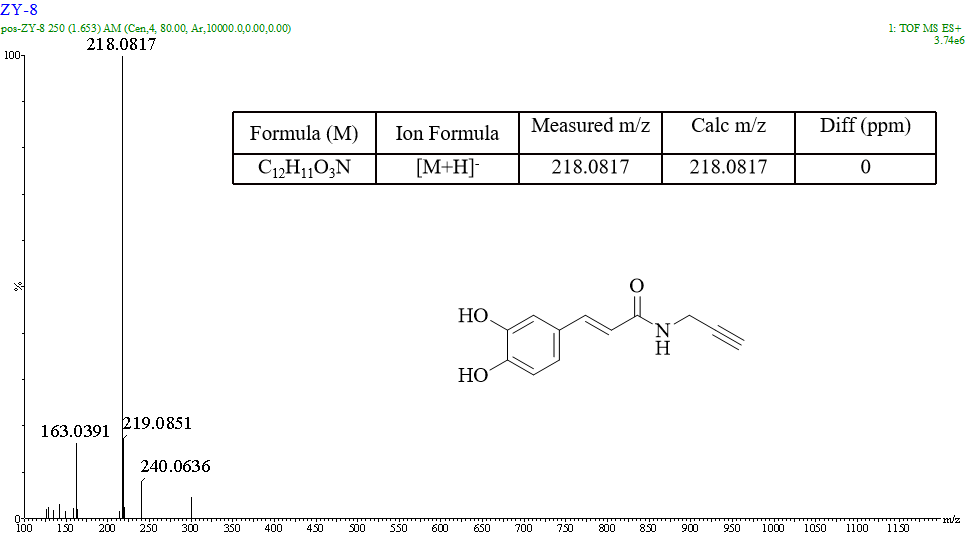
^

Fig. S4. HRMS spectrum of caffeic acid probe


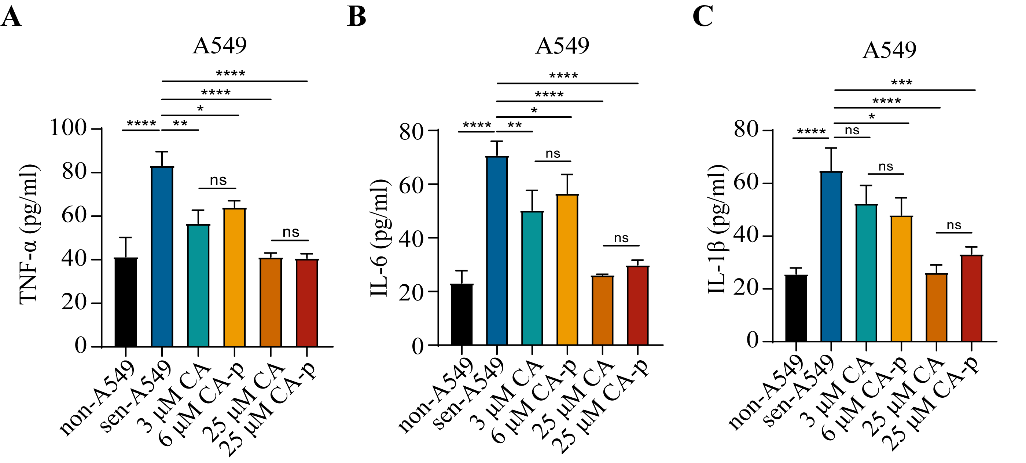


Fig. S5. The concentration changes of TNF-α (A), IL-6 (B) or IL1β (C) in the culture medium of non-senescent or senescent A549 cells after 25 μM CA or its probe treatment, n=3.

Data are mean ± s.e.m. One-way ANOVA test was used. *P<0.05, **P<0.01, ***P<0.001, ****P<0.0001. ns means no significance.


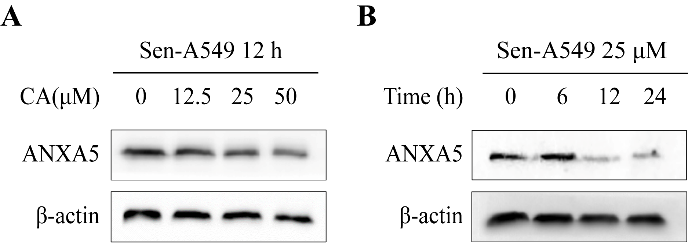


Fig. S6. Detection of ANXA5 protein levels in senescent A549 cells treated with increasing concentrations of CA by western blot.


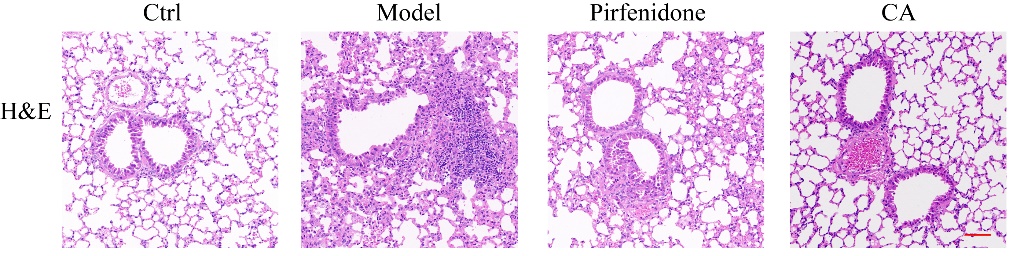


Fig. S7. Representative HE staining of lungs from model mice after CA or pirfenidone treatment. Scale bar, 100 μm.


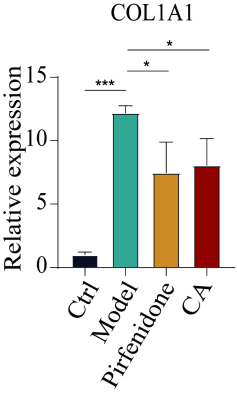


Fig. S8. CA decreased the expression of *Col1a1* at mRNA level in lungs. Data are the mean ± s.e.m. One-way ANOVA test was used. **P* < 0.05, ****P* < 0.001.


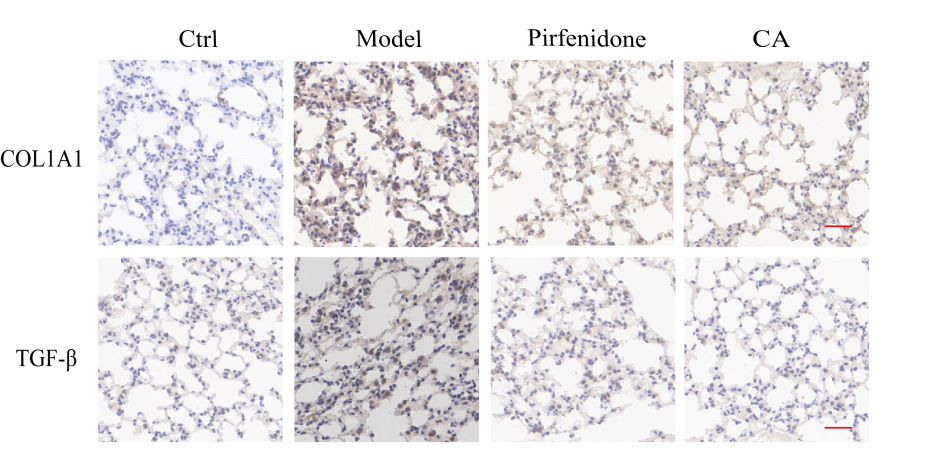


Fig. S9. The expression of COL1A1 and TGF-β was inhibited in CA-treated mice lungs by IHC. Scale bar, 100 μm.


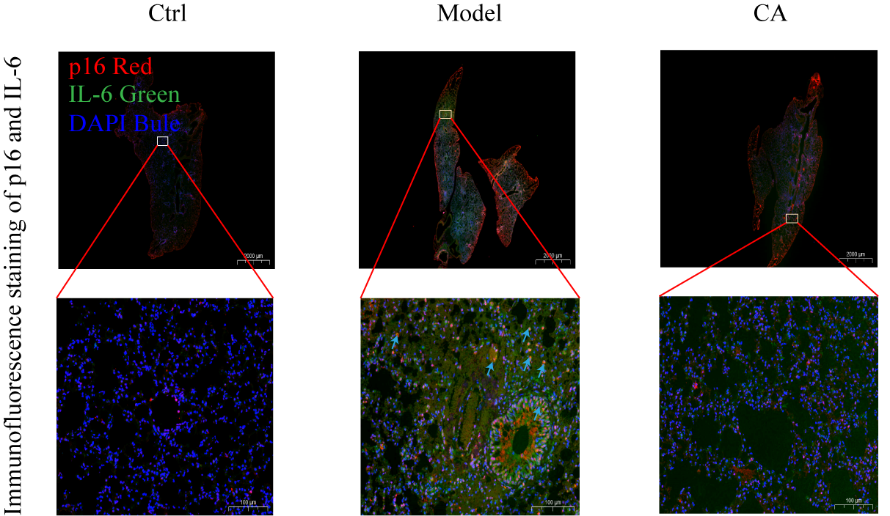


Fig. S10. CA reduced the expression of IL-6 in p16-positive senescent cells in lung slices from model mice as detected by immunofluorescence. The arrows indicated the p16 and IL-6 positive cells. Scale bar of upper images, 2000 μm. Scale bar of upper images, 100 μm.


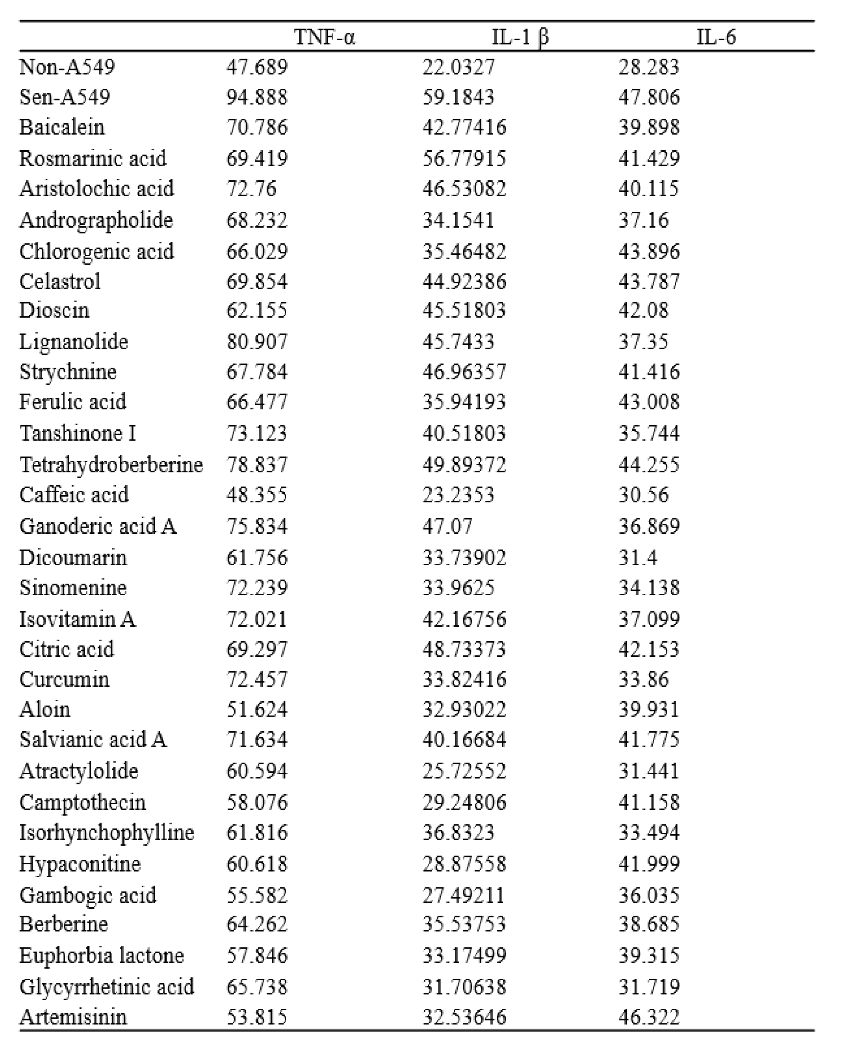


Table S1 Results of screening senomorphics from natural products
